# Maize and ancient Maya droughts

**DOI:** 10.1038/s41598-022-26761-3

**Published:** 2022-12-23

**Authors:** Gerald A. Islebe, Nuria Torrescano-Valle, Mirna Valdez-Hernández, Alicia Carrillo-Bastos, Alejandro A. Aragón-Moreno

**Affiliations:** 1 El Colegio de la Frontera Sur, Unidad Chetumal, Chetumal, Mexico; 2 Tecnológico Nacional de México / IT de Chetumal, Chetumal, Mexico

**Keywords:** Palaeoclimate, Palaeoecology, Climate-change adaptation

## Abstract

The ancient Maya culture of Mesoamerica shaped landscapes for centuries, in an area where maize (*Zea* *mays*) cultivation is considered a fundamental crop in the diet of present and ancient Mesoamerican cultures. Pollen records from sites with different environmental and climatic conditions of the Yucatán Peninsula (Mexico) and Peten (Guatemala) evidence a clear relationship between increased maize pollen and periods of reduced precipitation caused by El Niño Southern Oscillation (ENSO) while moist periods are characterized by low maize pollen presence. ENSO conditions were not evenly distributed across the Yucatán Peninsula, and regional droughts vary according to regional climate and geographical conditions. Our results indicate a strong relationship of increased maize and tropical forest decrease with dry periods, while the Late Preclassic Humid Period (ca. 500–200 BCE) is characterized by the absence of maize pollen. The dry Late Preclassic (300 BCE-250 CE) was a key period for increased maize production, suggesting a new conceptualization of maize. Maize changed from a basic diet crop to a pragmatic product to face adverse environmental conditions.

## Introduction

The flourishing of the ancient Maya culture in Mesoamerica during almost 3000 years is traditionally related to effective agricultural practices, cultural development, and scientific advances in mathematics and astronomy^1,2^. Ancient Maya cultural development can be divided into four periods: Early, Middle and Late Preclassic (1500 BCE to 250 CE), and Early to Late Classic (250 to 1200 CE)^3^.

The Maya experienced a societal and political disruption between 900 and 1100 CE under drying climate conditions that complicated a complete understanding of the causes and process of its demise^4,5^. Sufficient and continuous food supply was required since the Preclassic period to support permanent societal development. With the need for a permanent food supply, *Zea* *mays* is considered the most important crop of the ancient Maya culture^2^. Maya agricultural knowledge has been described as a legacy of ancestral agricultural practices^6^, with more than 600 plant species identified with specific uses^7,8^.

There is increasing knowledge that ancient Maya could manage the landscape as a forest garden on a local to regional scale^9–11^. Beach et al.^12^ called Mayacene the time period from 3000 to 1000 cal year BP (1050 BCE–950 CE), indicating that ancient Maya transformed the landscape and hydrological system on a larger regional scale. This change included the transformation of wetlands for agricultural use. Pohl et al.^13^ report the use of maize from wetlands of northern Belize around 3000 BCE and from 2500 BCE onwards, intensified maize agriculture. For the northern Yucatan Peninsula, in the Yalahau region, the wetlands were managed with dikes and dams^14^.

The effect of past climate change on ancient Maya, their cultural adaptation, and environmental resilience is under scrutiny, as most of the paleoecological research has focused on Late Classic Maya droughts. The ancient Maya culture adapted to changing environmental conditions and under different environmental conditions^15^. Many paleoclimate studies have revealed the effect of climate variability on ancient Maya culture and environment, and several other studies also suggest that drought periods have played a major role in their cultural development and demise^16,17^. Wet periods, with alternating short dry phases and protracted droughts, have been identified in the wider Maya region during the last 4000 years^16,18^, mainly driven by latitudinal shifts of the Intertropical Convergence Zone^5^. Those studies also recognize the impact of El Niño Southern Oscillation (ENSO) on climate dynamics and ecosystems^4,5,16,19–21^. The effect of present and past ENSO on southern Mexico and Central America is known from several studies^22,23^. A geochemical study from Lago Puerto Arturo in northern Guatemala suggested that dry environmental conditions favored agriculture in the Late Holocene^24^. Conversely, wetter conditions during the Preclassic inhibited the development of intensive maize agriculture until ca 400 BCE^25^.

More recently, ENSO during 2016 caused reduced precipitation and physiological stress conditions in trees of the Yucatan Peninsula (YP) area along a precipitation gradient, with tree species from the driest site more affected compared to tree species from the wettest sites^26^.

Evidence of maize cultivation since the early Preclassic is available from several pollen records^25^, hence the question remains on how droughts interacted with vegetation and land-use change during the Preclassic and Classic periods. A detailed look into paleoecological data since the Early Preclassic could improve the understanding of maize used by the ancient Maya in periods of reduced precipitation.

We hypothesize that under drier climatic conditions increased presence of maize pollen is detected in the pollen records. Hence, under humid climate conditions, the presence of maize pollen will be lower. Diatom and pollen records from Lake Tuspan^25^, and Rio Hondo^20^ indicate a shift to wet climate conditions that prevailed between 1000 and 850 BCE. Rosenswig et al.^27^ consider this period, around 1000 cal BCE, to be essential for cultural development, when maize increased its importance for ancient cultures in Mesoamerica. Rosenswig et al.^27^ do not infer this with paleoecological change, around 1000 cal BCE, while this date indicates a key period for maize, after a previous continuous drying trend between 4500 and 2800 cal year BP (2550—850 BCE) in Mesoamerica. After 3500 cal year BP (1550 BCE), an opening of the landscape can be observed in several paleoecological records of the Yucatan Peninsula. Climate-driven deforestation preceded maize production in the Peten-Itza area around 1000 cal BCE^28^, but is not conclusive for all areas of the Yucatan Peninsula.

## Results

### Early to Middle Preclassic (1500–600 BCE)

For the Early and Middle Preclassic, our northern and driest study sites (Ria Lagartos 1 and 2) evidence increasing maize presence with stronger ENSO activity around 1300–1100 BCE, 1000–800 BCE, 750–600 BCE^29,30^. Droughts of the Middle Preclassic period exhibit regional differences within the ancient Maya distribution area. The Early and Middle Preclassic transition evidenced geographical variability, showing that the wettest site (Peten-Itza) presents a negative Pearson correlation of Peten-Itza r(30) = −0.48, p = 0.01 between tropical forest pollen and ENSO. Dry climatic conditions in the eastern Yucatán Peninsula were detected between 900 and 600 BCE, and other areas of the Maya lowlands^20^, which fall within the 2800 cal BP event^31^. The Silvituc core exhibits a significant positive relation between tropical forest (r = 0.57), disturbance vegetation (r = 0.59), maize (r = 0.63) and ENSO, and fits within the time frame between 800 to 600 BCE of human occupation during this dry period. The Ria Lagartos sites showed a negative correlation between maize/tropical pollen and ENSO (Table 1).

**Table 1:** Matrix of Pearson correlation coefficients.

	Tropical forest/ENSO	Disturbance vegetation/ENSO	*Zea* *mays*/ENSO
**Early Preclassic**			
Peten-Itza	0.48	0.43	0.18
Rio Hondo	−0.131	0.17	0.29
Ria Lagartos 1	−0.04	0.08	0.08
Ria Lagartos 2	0.049	−0.03	−0.18
Silvituc	−0.293	−0.37	−0.34
Chumpich	−0.177	−0.38	−0.37
**Middle Preclassic**			
Peten-Itza	0.37	−0.37	−0.37
Rio Hondo	−0.27	0.05	0.05
Ria Lagartos 1	0.19	0.16	-0.13
Ria Lagartos 2	−0.13	0.06	-0.28
Silvituc	0.57*	0.59*	0.63*
Chumpich	0.36	−0.47	0.16
**Late Preclassic**			
Peten-Itza	−0.21	0.1	−0.12
Rio Hondo	0.99	−0.2	0.84
Ria Lagartos 1	0.27	0.1	0.1
Ria Lagartos 2	0.31	−0.29	−0.05
Silvituc	0.18	−0.69	0.05
Chumpich	0.2	0.33	0.33
**Early Classic**			
Peten-Itza	−0.46*	−0.46*	
Rio Hondo			
Ria Lagartos 1	−0.12	−0.35	−0.14
Ria Lagartos 2	0.49*	−0.6*	0.12
Silvituc	0.12	0.42	0.12
Chumpich	0.01	−0.28	−0.1
**Late Classic**			
Peten-Itza	−0.3	−0.27	−0.24
Rio Hondo			
Ria Lagartos 1	−0.22	0.27	0.46
Ria Lagartos 2	−0.89	−0.04	−0.03
Silvituc	−0.12	0.05	−0.02
Chumpich	−0.25	0.06	−0.02

*Significant correlation with 95% confidence interval (2 tailed significance).

### Middle to Late Preclassic transition (600 BCE-250 CE)

Between 550 and 200 BCE, a humid period is recorded, known as the Late Preclassic Humid Period (LPHP)^18^, which was identified in our sediment cores as a period with few maize pollen and enhanced values of pollen classified as tropical forest elements. Estimated mean precipitation was up to 20% higher during the LPHP compared to present-day values in the eastern YP^32^. The Pearson correlation coefficient was calculated for the relationship between tropical pollen forest taxa and ENSO. For this given time period, no correlation was detected, and expected given the conditions of increased precipitation levels.

After the LPHP, the calculated Pearson correlation coefficients indicated a correlation of ENSO with the wet sites of the southeastern Yucatán Peninsula, Rio Hondo and Silvituc (Rio Hondo r(3) = 0.84, p = 1; Silvituc r(22) = 0.37, p = 1; respectively). The northern dry sites showed no correlation, nor the wetter Peten-Itza site. Maize presence started to increase in our pollen records after climate-driven forest reduction at Ria Lagartos, Chumpich, Peten-Itza, Silvituc, and Rio Hondo during the late Preclassic around 250–1 BCE. Pollen of the tropical forest taxa of the Rio Hondo core correlated strongly with ENSO r(3) = 0.9, p = 1. However, we are aware of the low number of data points for this core in this time frame.

### Early Classic (250–500 CE)

During the Early Classic period, tropical forest elements of all sites showed a negative correlation with ENSO, except for lake Silvituc. This time frame fits well with the first abandonment of the Silvituc area during the Early Classic, and the increased presence of pollen from secondary vegetation (Torrescano-Valle & Islebe, 2015) as well as the generalized maize pollen reduction in all other pollen records.

### Late Classic to Post Classic (500–1200 CE)

During the Late Classic, the northern dry site shows a positive correlation between maize/ENSO (Ria Lagartos 1 r(15) = 0.46, p = 0.6). This fits well with the increased drought reported from the northern Yucatán Peninsula during the Late Classic. The other sites reported in this study exhibit a negative maize/ENSO correlation, as these areas were abandoned after 800 CE.

## Discussion

The increase of maize presence, during Early and Middle Preclassic, is observed following an increasing drying trend before 3500 cal year BP (1550 BCE). Maize increased its presence in northern Mesoamerica after 1000 BC^27^, which denotes increased land-use change near water bodies in this region (Fig. 3). Climate-induced deforestation is evidenced at 3000 cal year BP from Lake Peten-Itza^28^ and Lake Silvituc^33^, where the opening of the landscape was favored by the reduced precipitation.

The Late Preclassic Humid Period (LPHP), with its absence of maize in the pollen records, indirectly points out that other edible plant resources were available. This period also evidences increased values of tropical forest pollen taxa in many paleoecological records of the Maya lowlands^20,24,25,33^. After the LPHP, maize increased in all records, especially during the Late Preclassic drought (150–250 CE), when maize strongly increased its presence in the Ria Lagartos, Peten-Itza, Silvituc, and Chumpich pollen records. Then maize presence dropped again after 250 CE, as regional precipitation began to stabilize^32^. The Late Preclassic drought evidenced a precipitation decrease of at least 20% in most parts of the YP, compared to the previous period, and was the strongest drought of the last 2000 years of the northern Yucatan Peninsula. This has also been observed in the current data of ENSO 2016. which caused a decrease in precipitation of up to 34% in the north of the Yucatan Peninsula^26^. Lake Peten Itza is the most humid of all sites, with a mean annual precipitation of ca. 1600 mm, the reduced precipitation could have caused less stresson tropical forests. In the Preclassic period, a more intensified form of agriculture appeared as an adaptation to increased population and reduced precipitation levels^34,35^. Intensified agricultural activity was achieved by terracing, raised fields, irrigation, and canals among others^35^. Maize could therefore be used with improved agricultural intensities in response to regional droughts and climate-driven deforestation or human land-use change. Outside the ancient Maya core area, from Costa Rica, Kerr^36^ presents similar evidence of increasing maize agriculture during periods of drought. Maize agriculture at Laguna Bonillita peaked at 150 CE during the late Preclassic drought.

After the Late Preclassic drought, the mean precipitation increases match again with the drop in maize pollen percentages in all cores (Fig. 3). After 250 CE complex processes of societal change were observed in Maya societies with increasing population growth in the Central Maya lowlands until the Terminal Classic^37^.

Pollen spectra from the Classic period evidence the reduced presence of maize pollen after ca. 300 CE, providing indirect evidence that major agroforestry practices were not mainly dependent on maize. Taxa of disturbance vegetation are continuously present in the Classic period, dropping after the Terminal Classic period (Fig. 3). Droughts impacted selectively in the Yucatán Peninsula, considering the geographical distribution of the studied pollen records. Timing, geographical distribution, and response to environmental factors evidence a mosaic of paleoecological and paleoclimatological change. Present-day analysis of climatic anomalies of the Yucatán Peninsula shows that five different clusters of precipitation anomalies exist^38^, the spatial effect of these anomalies on the vegetation is especially distinguishable under ENSO conditions with reduced precipitation^26^. All the six pollen records fall within these clusters of precipitation anomaly. The fossil pollen spectra reflect drier environmental conditions at given periods and have been detected on a regional scale^30,39^. At present, the southeastern and southwestern parts of the Yucatán Peninsula are the wettest areas of this region and maize benefitted from reduced precipitation in those regions. The Late Preclassic drought had its strongest influence combined with human land-use change on vegetation in the southeastern and southwestern part (Rio Hondo and Silvituc cores).

The fossil pollen maize data and their relationship with alternating precipitation levels indicated increased maize production after 300–200 BCE as a response to regional dry climatic conditions, local environmental conditions, and tropical forest disturbance. The ancient Maya culture did not depend exclusively on maize, as many other edible plant species were available^8^ The importance of maize during dry stages of the Preclassic period, after climate-induced forest openings, was essential. We agree with Tuxill et al.^40^ that maize was a famine breaker during drought periods. The geographical extension of droughts and their effect on tropical forests, and maize agriculture, is a key factor to understand how ancient Maya culture developed.

## Methods

**Figure 1:**
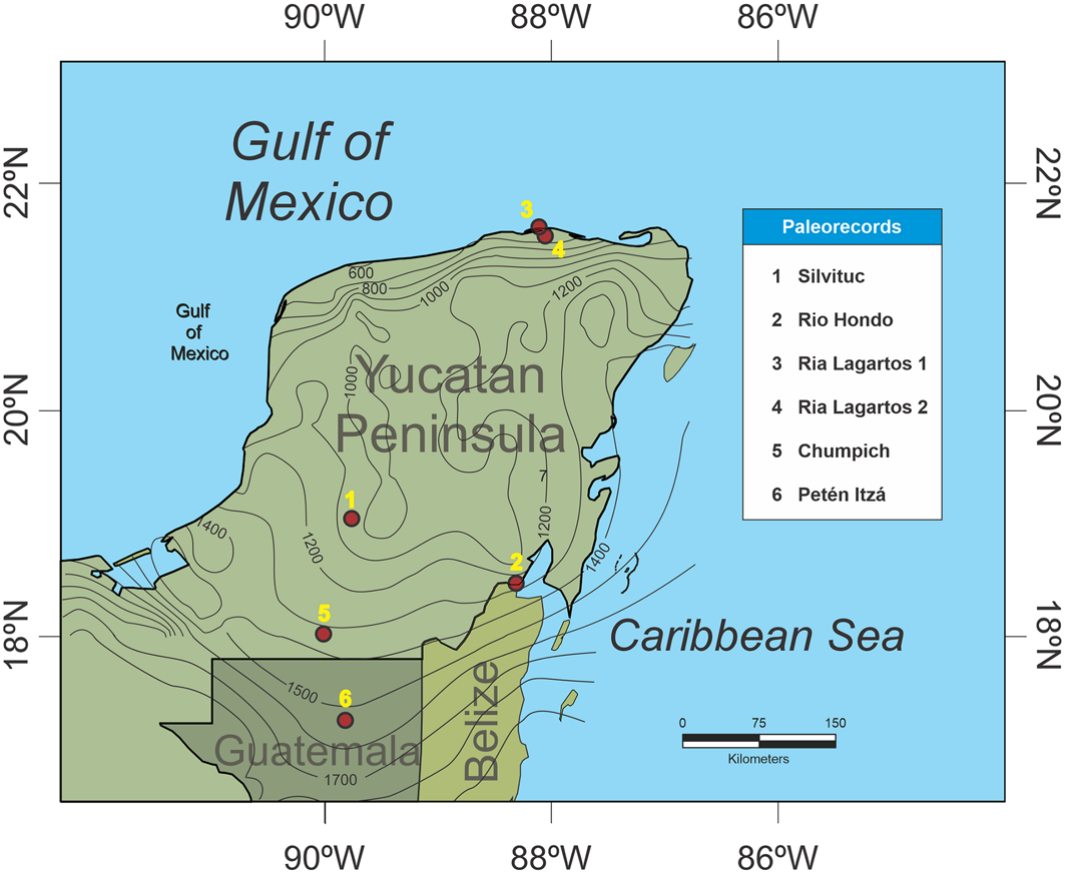
Core locations in the Yucatan Peninsula and Northern Guatemala. Precipitation isohyets showing the present precipitation values. The generated with QGIS versión 3.22.11^58^ (https://qgis.org) and isohyets were drawn based on the precipitation map from INEGI^59^.

In this study, we analyzed the presence of fossil maize pollen, from several sediment cores of the Yucatan Peninsula (YP) and northern Guatemala, related to ENSO variability during the last ~ 4000 years (Fig. 1). The data analysis includes the numerical relationship of past ENSO signals compared to fossil maize pollen presence. Six sediment cores located along a precipitation gradient (800–1500 mm/year) in dry tropical forest areas in the Yucatan Peninsula and northern Guatemala were analyzed. Chronological control of all cores was achieved by AMS radiocarbon dating and original calibration data. All methods were carried out in accordance with relevant institutional, national, and international guidelines and legislation. The following cores were used (See Supplementary Information for extended data of the six pollen records):

1. Ria Lagartos 1, located in the northern YP. The area has a mean annual precipitation of 500 mm, and a mean annual temperature of 26ºC. This core site has nearby seasonal flooded dry forest and mangroves. A 1.90 m length core was taken at this site^29^.
2. Ria Lagartos 2, refer to Ria Lagartos 1 for site information^30^.
3. Lake Silvituc is in the southern YP in the state of Campeche. The mean annual precipitation of the area is 1300 mm/year, with a mean annual temperature of 26 ºC. A sediment core of 1.35 m was retrieved. The dominant vegetation type at present is seasonal dry tropical forest^33^.
4. Rio Hondo is in the southeastern part of the YP in the state of Quintana Roo. The retrieved sediment core is 8 m long, located in an area of 1300–1400 mm of annual precipitation, with a mean annual temperature of 26.5 ºC. The vegetation consists of a mixture of mangrove and low-statured tropical forest^20^.
5. Chumpich Lake is in the southern part of the YP, the nearest archaeological site corresponds to Uxul, which is ~ 50 km from Calakmul and ~ 30 km from El Mirador. The lake is 1.7 km length and 1.3 km wide, surrounded by a low-statured flooded forest that connects it with other nearby lakes such as San Felipe and the Mirador basin (Wahl et al., 2007). Precipitation ranges from 1300 to 1500 mm per year. The vegetation is mainly composed of tropical medium forests, characterized by the presence of *Brosimum* *alicastrum*, *Manilkara* *zapota*, *Talisia* *olivaeformis*, and *Pimenta* *dioica*. The low forest presents species like *Haematoxylum* *campechianum,* *Metopium* *brownei,* *Bucida* *buceras,* *Cladium* *jamaicense*, and Cyperaceae^41^.
6. Lake Petén-Itza is in northern Guatemala, in the Peten district. For lake characteristics see Islebe et al.^17^. The core was 5.45 m long. Mean annual precipitation is around 1600 mm and with a mean annual temperature of 25 ºC. Vegetation around the lake consists mainly of tropical forest with species from the Moraceae and Fabaceae families, among others.

In Table 2, all taxa from the six pollen records are presented.

**Table 2:** Taxa from the six pollen records.

Taxa	Vegetation	Site
*Alseis* *yucatanensis*	Tropical forest	Ria Lagartos1
Anacardiaceae	Tropical forest	Peten Itza, Rio Hondo, Ria Lagartos1, Ria Lagartos2, Silvituc, Chumpich
Apocynaceae	Tropical forest	Peten Itza, Rio Hondo, Ria Lagartos1, Ria Lagartos2, Silvituc, Chumpich
Araliaceae	Tropical forest	Peten Itza, Rio Hondo
Arecaceae	Tropical forest	Rio Hondo, Ria Lagartos1, Ria Lagartos2, Chumpich, Silvituc
*Bauhinia* sp.	Tropical forest	Peten Itza
Bignoniaceae	Tropical forest	Peten Itza, Rio Hondo, Ria Lagartos1, Ria Lagartos2, Silvituc, Chumpich
Bombacaceae	Tropical forest	Peten Itza, Rio Hondo
Boraginaceae	Tropical forest	Peten Itza, Ria Lagartos1, Ria Lagartos2, Chumpich
*Borreria* sp.	Tropical forest	Peten Itza, Silvituc
*Brosimum* *alicastrum*	Tropical forest	Peten Itza, Rio Hondo, Ria Lagartos1, Ria Lagartos2, Silvituc, Chumpich
*Bucida* sp.	Tropical forest	Peten Itza, Ria Lagartos2
*Bursera* *simarouba*	Tropical forest	Peten Itza, Rio Hondo, Ria Lagartos1, Ria Lagartos2, Silvituc
Burseraceae	Tropical forest	Peten Itza
*Byrsonima* sp.	Tropical forest	Peten Itza, Chumpich, Ria Lagartos1
*Caesalpinia* sp	Tropical forest	Chumpich
*Ceiba* sp.	Tropical forest	Chumpich
*Chlorophora*	Tropical forest	Peten Itza
*Clusia* sp.	Tropical forest	Peten Itza
Clusiaceae	Tropical forest	Peten Itza
*Coccoloba* sp.	Tropical forest	Peten Itza
Combretaceae	Tropical forest	Peten Itza, Rio Hondo, Ria Lagartos2, Silvituc
*Cordia* sp.	Tropical forest	Peten Itza, Ria Lagartos2
*Dalbergia* *glabra*	Tropical forest	chumpich
*Drypetes* sp.	Tropical forest	Peten Itza, Rio Hondo, Ria Lagartos2
Erythroxylum	Tropical forest	Peten Itza
*Euphorbia* sp.	Tropical forest	Peten Itza, Silvituc
Euphorbiaceae	Tropical forest	Peten Itza, Rio Hondo, Ria Lagartos1, Ria Lagartos2, Silvituc, Chumpich
Fabaceae	Tropical forest	Peten Itza, Rio Hondo, Ria Lagartos1, Ria Lagartos2, Silvituc, Chumpich
*Ficus* sp.	Tropical forest	Peten Itza, Rio Hondo, Ria Lagartos1, Ria Lagartos2, Silvituc, Chumpich
*Guettarda* *combsi*	Tropical forest	Peten Itza, Rio Hondo, Silvituc, Chumpich
*Gymnanthes* sp.	Tropical forest	Ria Lagartos1
*Hampea* *trilobata*	Tropical forest	Peten Itza, Silvituc
*Haematoxylum* *campechianum*	Tropical forest	Rio Hondo, Ria Lagartos2, Chumpich
*Hedyosmum* sp.	Tropical forest	Peten Itza
*Lonchocarpus* sp	Tropical forest	Chumpich
Malpighiaceae	Tropical forest	Peten Itza, Rio Hondo, Ria Lagartos1, Ria Lagartos2, Silvituc, chumpich
Meliaceae	Tropical forest	Peten Itza, Rio Hondo, Ria Lagartos2, Silvituc, Chumpich
*Metopium* *brownei*	Tropical forest	Peten Itza, Rio Hondo, Ria Lagartos1, Ria Lagartos2
Moraceae	Tropical forest	Peten Itza, Rio Hondo, Ria Lagartos1, Ria Lagartos2, Silvituc
Myrtaceae	Tropical forest	Peten Itza, Rio Hondo, Ria Lagartos1, Ria Lagartos2, Silvituc, chumpich
*Oreopanax* sp.	Tropical forest	Peten Itza
*Pachira* *aquatica*	Tropical forest	Rio Hondo
*Pithecellobium* sp.	Tropical forest	Rio Hondo
Polygonaceae	Tropical forest	Peten Itza, Silvituc, Chumpich, Ria Lagartos2
*Pouteria* sp.	Tropical forest	Peten Itza, Rio Hondo, Ria Lagartos1, Ria Lagartos2, Silvituc, chumpich
Rubiaceae	Tropical forest	Peten Itza, Rio Hondo, Ria Lagartos1, Silvituc, Chumpich
Sapindaceae	Tropical forest	Peten Itza, Rio Hondo, Ria Lagartos1, Ria Lagartos2, Silvituc, chumpich
*Sapium* sp.	Tropical forest	Peten Itza, Silvituc, Chumpich
Sapotaceae	Tropical forest	Peten Itza, Rio Hondo, Ria Lagartos1, Ria Lagartos2, Silvituc, chumpich
*Spondias* sp.	Tropical forest	Peten Itza, Ria Lagartos2, Silvituc, Chumpich
*Swartzia* sp.	Tropical forest	Peten Itza, Ria Lagartos2
*Terminalia* sp.	Tropical forest	Peten Itza
Urticales	Tropical forest	Peten Itza
*Zuelania* sp.	Tropical forest	Peten Itza
*Acacia* sp.	Disturbance vegetation	Peten Itza, Rio Hondo, Ria Lagartos1, Ria Lagartos2, Chumpich
*Acalypha* sp.	Disturbance vegetation	Peten Itza
Acanthaceae	Disturbance vegetation	Peten Itza, Rio Hondo, Ria Lagartos1, Ria Lagartos2, Chumpich
*Althernanthera* sp.	Disturbance vegetation	Rio Hondo, Chumpich
*Alchornea* sp.	Disturbance vegetation	Peten Itza
Amaryllidaceae	Disturbance Vegetation	Peten Itza, Ria Lagartos2
*Ambrosia* sp.	Disturbance vegetation	Peten Itza
Asteraceae	Disturbance vegetation	Peten Itza, Rio Hondo, Ria Lagartos1, Ria Lagartos2, Chumpich
*Bravaisia* *tubiflora*	Disturbance vegetation	Peten Itza, Rio Hondo, Silvituc, Chumpich
*Canavalia* sp.	Disturbance vegetation	Peten Itza
*Cecropia* sp.	Disturbance vegetation	Peten Itza, Rio Hondo, Ria Lagartos1, Ria Lagartos2
*Celtis* sp.	Disturbance vegetation	Peten Itza, Rio Hondo, Ria Lagartos1, Ria Lagartos2, Chumpich
Chenopodiaceae/Amaranthaceae	Disturbance vegetation	Peten Itza, Rio Hondo, Ria Lagartos1, Ria Lagartos2, Chumpich
*Cleome* sp.	Disturbance vegetation	Ria Lagartos2
*Cnidoscolus* sp.	Disturbance vegetation	Peten Itza, Rio Hondo, Ria Lagartos2
Convolvulaceae	Disturbance vegetation	Peten Itza, Rio Hondo, Ria Lagartos1, Ria Lagartos2, Chumpich
*Croton* sp.	Disturbance vegetation	Peten Itza, Ria Lagartos1, Ria Lagartos2, Silvituc, Chumpich
Cucurbitaceae	Disturbance vegetation	Peten Itza, Rio Hondo, Ria Lagartos1, Ria Lagartos2, Silvituc, Chumpich
Flacourtiaceae	Disturbance vegetation	Peten Itza
*Jaquemontia* sp.	Disturbance vegetation	Peten Itza, Ria Lagartos1, RiaLagartos2
*Justicia* *campechiana*	Disturbance vegetation	Chumpich
Labiateae	Disturbance vegetation	Peten Itza, Ria Lagartos1, RiaLagartos2
Loranthaceae type	Disturbance vegetation	Peten Itza
Malvaceae	Disturbance vegetation	Peten Itza, Rio Hondo, Ria Lagartos1, Ria Lagartos2, Silvituc, chumpich
Mimosoideae-*Acacia*	Disturbance vegetation	Peten Itza, Rio Hondo, Silvituc
*Myrica* sp.	Disturbance vegetation	Ria Lagartos1, Ria Lagartos2, Silvituc, Chumpich
Onagraceae	Disturbance vegetation	Peten Itza, Silvituc
Passiflorae	Disturbance vegetation	Peten Itza, Silvituc
*Pilea* sp.	Disturbance vegetation	Peten Itza
Piperaceae	Disturbance vegetation	Peten Itza
Poaceae	Disturbance vegetation	Peten Itza, Rio Hondo, Ria Lagartos1, Ria Lagartos2, Silvituc, Chumpich
*Satureja* sp.	Disturbance vegetation	Peten Itza
Solanaceae	Disturbance vegetation	Peten Itza, Rio Hondo, Ria Lagartos1, Ria Lagartos2, Chumpich
*Trema* sp.	Disturbance vegetation	Peten Itza
Ulmaceae	Disturbance vegetation	Peten Itza, Silvituc, Chumpich
Verbenaceae	Disturbance vegetation	Ria Lagartos1, Ria Lagartos2

### Pollen extraction

Acetolysis was used to extract pollen of all sediment cores^42^, and for detailed methods, we refer to the original publications. Original pollen diagrams are available in their respective references. The identified *Zea* *mays* pollen grains of all cores ranged between 65 and 90 microns. Additional pollen slides from the different cores were scanned for *Z.* *mays* to avoid missing grains. *Zea* *mays* was identified using size and pore morphology, and in doubt phase contrast was used.

### Zea mays

Among the main food sources used by the Maya culture during the Preclassic and Classic periods were *Brosimum* *alicastrum* and *Zea* *mays*^43,44^. *B.* *alicastrum* trees (breadnut) are common around milpa areas^45^ and seeds are used up to present time by Maya farmers^46^. *Brosimum* *alicastrum* may have been an important food source for ancient Maya. The management of breadnuts is proved by charcoal analysis from northern Guatemala^47^, providing extensive use of this tree species.

Yucatec Mayans selected maize varieties with different cycles, namely, a short cycle maize of 7 to 10 weeks and a long cycle maize of 12 and 16 weeks^40^. The duration of the cycle allows maize to avoid the intra-seasonal drought that occurs during the rainy season. Since planting occurs at the beginning of the rainy season, intra-seasonal drought can concur with two crucial development stages and with higher demand for water, like the development of spikes or the growth of maize kernels on the cob^40^. For this reason, Maya have traditionally mixed different varieties of maize on the same plot. The short cycle maize varieties ensure the development of the spikes before the intra-seasonal drought and are known as nal t'eel, x-t'uup nal and x-mejen nal. Varieties with the long cycle guarantee higher grain production in case of a good rainy season and are known as X-nuuk nal, Ts'ı´it'it bakal, Bek'ech bakal^40^. The expected production for a traditional Maya milpa is 2.5 ton ha-1 per planting cycle^48^. While the production of *B.* *alicastrum* depends on the age, and trees of 25 years produce up to 19 ton year-1 of fresh seed^49^, equivalent to 4 ton year-1 of dry seed, with up to 80% of moisture content^50^. However, the impact of drought and rising temperatures on biomass production depends on how adapted the species are to water stress. Water use efficiency (WUE) showed the relationship between plant productivity and water use^51^. Under this view, maize has an advantage, as its productivity can increase without changing the rate of water use, achieving an increase in WUE^52^. Some maize crops may record a WUE of 5 μmol mol^−1^, while *B.* *alicastrum* presents a maximum WUE of 1.5 μmol mol^−1^ ^49^. In addition, cropping strategies can improve WUE. Increasing row count and planting density help reduce the effect on soil water evaporation and is an effective strategy in dry regions with variable rainfall episodes and high demand for evaporation^53^.

### Data analysis

#### Zea mays interpolation approach

**Figure 2:**
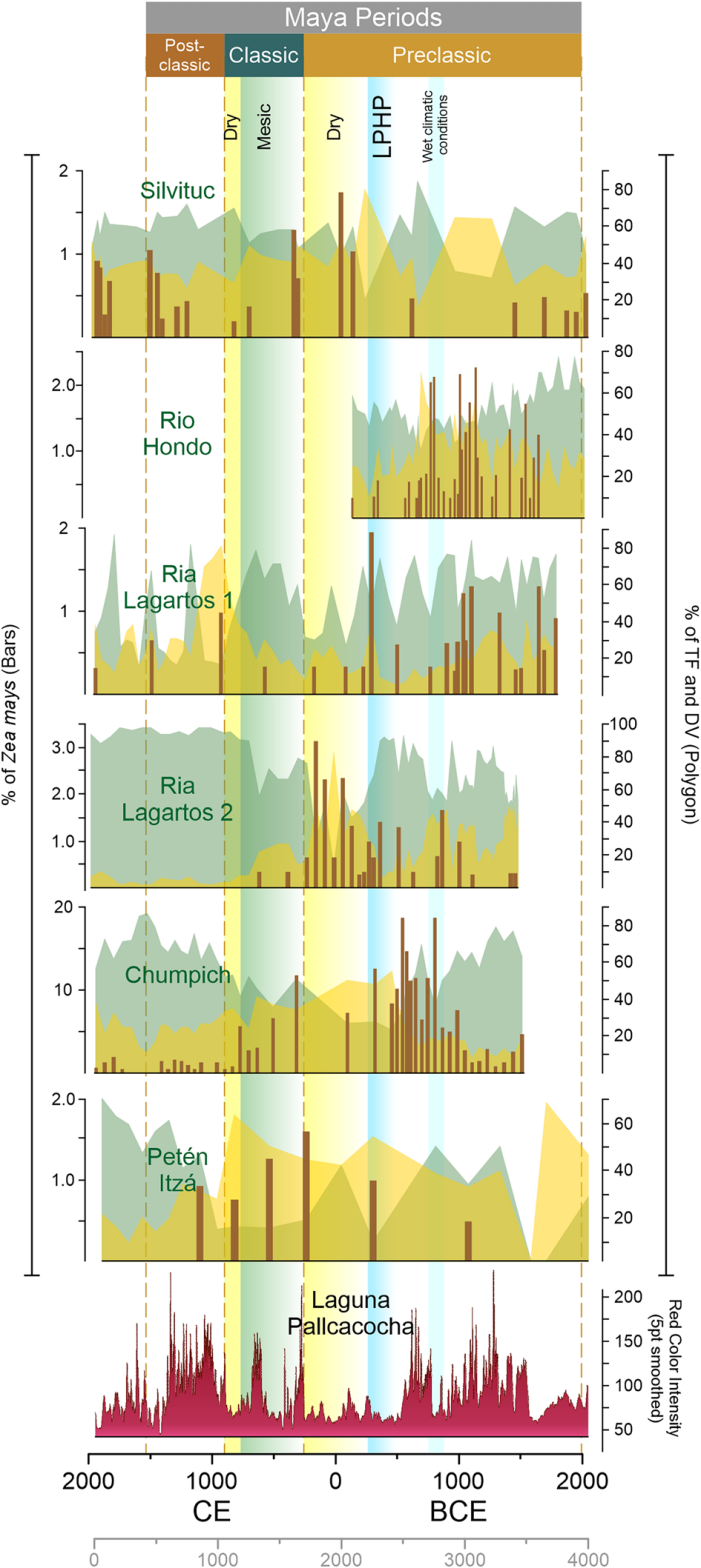
Pollen diagrams of cores in the Maya area of the YP. Tropical Forest (TF; shaded green) and disturbance vegetation (DV; shaded yellow) percentages along with *Zea* *mays* (brown bars) and smoothed Red Color Intensity in Laguna Pallcacocha^54^ as ENSO activity signal.

**Figure 3:**
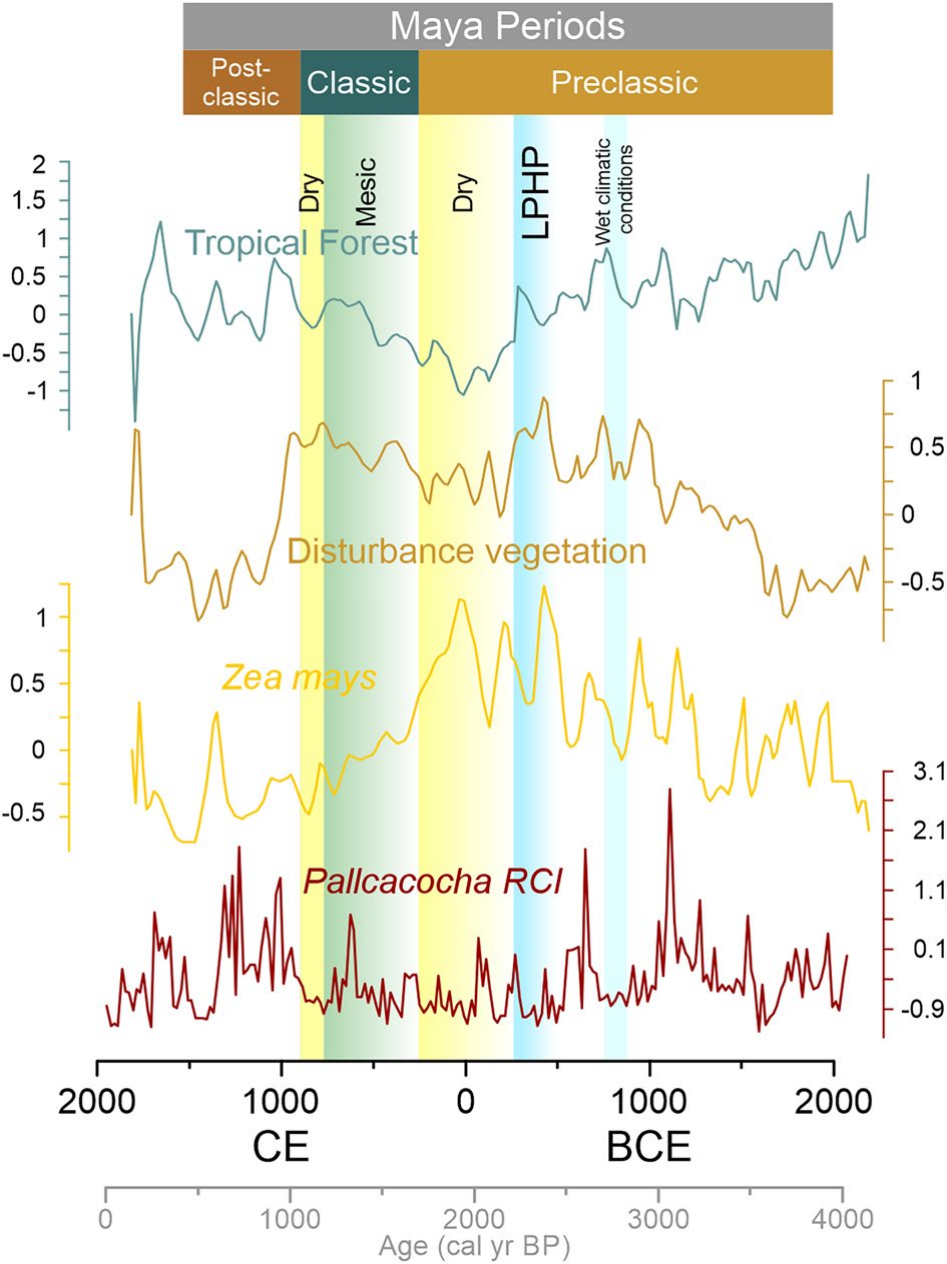
Average Z-scores values of fossil pollen groups: Tropical Forest, Disturbance vegetation, *Zea* *mays*, and Pallcacocha RCI (red color intensity) as ENSO Variability^54^. Periods of previously reported general climate variability are highlighted in columns.

The pollen counts of every site were categorized into the vegetation types: *Zea* *mays*, disturbance vegetation, and tropical forest (Fig. 2). Data standardization was made using the Z score function, to obtain comparable values between the pollen records of the sites Río Hondo, Ria Lagartos, Silvituc, Chumpich, Peten Itza, and ENSO variability in lake Pallcacocha (Red Color Intensity)^54^. To establish temporal correspondence between sites, R statistical software version 4.0.5 (R Core Team, 2021) was used, Interpolation function of “psych” package^55^ was applied together with “readxl”^56^, and “corrplot”^57^ packages. ENSO variability supplied the model with chronology, and a 20-year time interval was used to model interpolated values in each pollen record, keeping the original chronology of pollen records unchanged. Data from Laguna Pallcacocha which could not be paired was not used. The interpolation was made to obtain comparable pollen data between all sites, and under the same time frame, without losing pollen data. Computed output was compared to each original data curve, to validate the trend of the newly modeled vs the original data. After modeled pollen data of individual records were obtained, a composite data set of Z scores of the pollen data was graphed for each vegetation type in Fig. 3. Average pollen values of each vegetation type were visually comparable with ENSO variability. The Pearson correlation coefficients between vegetation types and ENSO were obtained for every site (Table 1). The correlation indexes were analyzed according to the Maya occupation periods, from the Early Preclassic to the Late Classic period. This time frame approximately corresponds to the last 3500 years (Supplementary information).

## Supplementary Information

Supplementary Information.

## Data Availability

The datasets used and/or analyzed during the current study available from the corresponding author on reasonable request.
