# Supplementary material for: Maize and ancient Maya droughts
Source: Sci Rep. 2022 Dec 23;12:22272. doi: 10.1038/s41598-022-26761-3 (PMC9789100; doi:10.1038/s41598-022-26761-3)
Supplement: Supplementary file 1 — Supplementary Information. [file 41598_2022_26761_MOESM1_ESM.docx]

Maize and ancient Maya droughts

**Gerald A. Islebe^1^, Nuria Torrescano-Valle^1^*, Mirna Valdez-Hernández^1^, Alicia Carrillo-Bastos^2^, Alejandro Aragón-Moreno^1^***

^1^El Colegio de la Frontera Sur, Unidad Chetumal, Mexico

^2^Tecnológico Nacional de México/ IT Chetumal, Mexico

*** Correspondence:**Alejandro A. Aragón-Moreno

[alejandro.aragon@ecosur.mx](mailto:alejandro.aragon@ecosur.mx)

Nuria Torrescano-Valle

[ntorresca@ecosur.mx](mailto:ntorresca@ecosur.mx)

**Extended Data Table 2. Taxa from the six pollen records**

| **Taxa** | **Vegetation** | **Site** |
| --- | --- | --- |
| *Alseis yucatanensis* | Tropical Forest | Ria Lagartos1 |
| Anacardiaceae | Tropical Forest | Peten Itza, Rio Hondo, Ria Lagartos1, Ria Lagartos2, Silvituc, Chumpich |
| Apocynaceae | Tropical Forest | Peten Itza, Rio Hondo, Ria Lagartos1, Ria Lagartos2, Silvituc, Chumpich |
| Araliaceae | Tropical Forest | Peten Itza, Rio Hondo |
| Arecaceae | Tropical Forest | Rio Hondo, Ria Lagartos1, Ria Lagartos2, Chumpich, Silvituc |
| *Bauhinia* sp. | Tropical Forest | Peten Itza |
| Bignoniaceae | Tropical Forest | Peten Itza, Rio Hondo, Ria Lagartos1, Ria Lagartos2, Silvituc, Chumpich |
| Bombacaceae | Tropical Forest | Peten Itza, Rio Hondo |
| Boraginaceae | Tropical Forest | Peten Itza, Ria Lagartos1, Ria Lagartos2, Chumpich |
| *Borreria* sp. | Tropical Forest | Peten Itza, Silvituc |
| *Brosimum alicastrum* | Tropical Forest | Peten Itza, Rio Hondo, Ria Lagartos1, Ria Lagartos2, Silvituc, Chumpich |
| *Bucida* sp. | Tropical Forest | Peten Itza, Ria Lagartos2 |
| *Bursera simarouba* | Tropical Forest | Peten Itza, Rio Hondo, Ria Lagartos1, Ria Lagartos2, Silvituc |
| Burseraceae | Tropical Forest | Peten Itza |
| *Byrsonima* sp. | Tropical Forest | Peten Itza, Chumpich, Ria Lagartos1 |
| *Caesalpinia* sp | Tropical Forest | Chumpich |
| *Ceiba* sp. | Tropical Forest | Chumpich |
| *Chlorophora* | Tropical Forest | Peten Itza |
| *Clusia* sp. | Tropical Forest | Peten Itza |
| Clusiaceae | Tropical Forest | Peten Itza |
| *Coccoloba* sp. | Tropical Forest | Peten Itza |
| Combretaceae | Tropical Forest | Peten Itza, Rio Hondo, Ria Lagartos2, Silvituc |
| *Cordia* sp. | Tropical Forest | Peten Itza, Ria Lagartos2 |
| *Dalbergia glabra* | Tropical Forest | chumpich |
| *Drypetes* sp. | Tropical Forest | Peten Itza, Rio Hondo, Ria Lagartos2 |
| Erythroxylum | Tropical Forest | Peten Itza |
| *Euphorbia* sp. | Tropical Forest | Peten Itza, Silvituc |
| Euphorbiaceae | Tropical Forest | Peten Itza, Rio Hondo, Ria Lagartos1, Ria Lagartos2, Silvituc, Chumpich |
| Fabaceae | Tropical Forest | Peten Itza, Rio Hondo, Ria Lagartos1, Ria Lagartos2, Silvituc, Chumpich |
| *Ficus* sp. | Tropical Forest | Peten Itza, Rio Hondo, Ria Lagartos1, Ria Lagartos2, Silvituc, Chumpich |
| *Guettarda combsi* | Tropical Forest | Peten Itza, Rio Hondo, Silvituc, Chumpich |
| *Gymnanthes* sp. | Tropical Forest | Ria Lagartos1 |
| *Hampea trilobata* | Tropical Forest | Peten Itza, Silvituc |
| *Haematoxylum campechianum* | Tropical Forest | Rio Hondo, Ria Lagartos2, Chumpich |
| *Hedyosmum* sp. | Tropical Forest | Peten Itza |
| *Lonchocarpus* sp | Tropical Forest | Chumpich |
| Malpighiaceae | Tropical Forest | Peten Itza, Rio Hondo, Ria Lagartos1, Ria Lagartos2, Silvituc, chumpich |
| Meliaceae | Tropical Forest | Peten Itza, Rio Hondo, Ria Lagartos2, Silvituc, Chumpich |
| *Metopium brownei* | Tropical Forest | Peten Itza, Rio Hondo, Ria Lagartos1, Ria Lagartos2 |
| Moraceae | Tropical Forest | Peten Itza, Rio Hondo, Ria Lagartos1, Ria Lagartos2, Silvituc |
| Myrtaceae | Tropical Forest | Peten Itza, Rio Hondo, Ria Lagartos1, Ria Lagartos2, Silvituc, chumpich |
| *Oreopanax* sp. | Tropical Forest | Peten Itza |
| *Pachira aquatica* | Tropical Forest | Rio Hondo |
| *Pithecellobium* sp. | Tropical Forest | Rio Hondo |
| Polygonaceae | Tropical Forest | Peten Itza, Silvituc, Chumpich, Ria Lagartos2 |
| *Pouteria* sp. | Tropical Forest | Peten Itza, Rio Hondo, Ria Lagartos1, Ria Lagartos2, Silvituc, chumpich |
| Rubiaceae | Tropical Forest | Peten Itza, Rio Hondo, Ria Lagartos1, Silvituc, Chumpich |
| Sapindaceae | Tropical Forest | Peten Itza, Rio Hondo, Ria Lagartos1, Ria Lagartos2, Silvituc, chumpich |
| *Sapium* sp. | Tropical Forest | Peten Itza, Silvituc, Chumpich |
| Sapotaceae | Tropical Forest | Peten Itza, Rio Hondo, Ria Lagartos1, Ria Lagartos2, Silvituc, chumpich |
| *Spondias* sp. | Tropical Forest | Peten Itza, Ria Lagartos2, Silvituc, Chumpich |
| *Swartzia* sp. | Tropical Forest | Peten Itza, Ria Lagartos2 |
| *Terminalia* sp. | Tropical Forest | Peten Itza |
| Urticales | Tropical Forest | Peten Itza |
| *Zuelania* sp. | Tropical Forest | Peten Itza |
| *Acacia* sp. | Disturbance Vegetation | Peten Itza, Rio Hondo, Ria Lagartos1, Ria Lagartos2, Chumpich |
| *Acalypha* sp. | Disturbance Vegetation | Peten Itza |
| Acanthaceae | Disturbance Vegetation | Peten Itza, Rio Hondo, Ria Lagartos1, Ria Lagartos2, Chumpich |
| *Althernanthera* sp. | Disturbance Vegetation | Rio Hondo, Chumpich |
| *Alchornea* sp. | Disturbance Vegetation | Peten Itza |
| Amaryllidaceae | Disturbance Vegetation | Peten Itza, Ria Lagartos2 |
| *Ambrosia* sp. | Disturbance Vegetation | Peten Itza |
| Asteraceae | Disturbance Vegetation | Peten Itza, Rio Hondo, Ria Lagartos1, Ria Lagartos2, Chumpich |
| *Bravaisia tubiflora* | Disturbance Vegetation | Peten Itza, Rio Hondo, Silvituc, Chumpich |
| *Canavalia* sp. | Disturbance Vegetation | Peten Itza |
| *Cecropia* sp. | Disturbance Vegetation | Peten Itza, Rio Hondo, Ria Lagartos1, Ria Lagartos2 |
| *Celtis* sp. | Disturbance Vegetation | Peten Itza, Rio Hondo, Ria Lagartos1, Ria Lagartos2, Chumpich |
| Chenopodiaceae/Amaranthaceae | Disturbance Vegetation | Peten Itza, Rio Hondo, Ria Lagartos1, Ria Lagartos2, Chumpich |
| *Cleome* sp. | Disturbance Vegetation | Ria Lagartos2 |
| *Cnidoscolus* sp. | Disturbance Vegetation | Peten Itza, Rio Hondo, Ria Lagartos2 |
| Convolvulaceae | Disturbance Vegetation | Peten Itza, Rio Hondo, Ria Lagartos1, Ria Lagartos2, Chumpich |
| *Croton* sp. | Disturbance Vegetation | Peten Itza, Ria Lagartos1, Ria Lagartos2, Silvituc, Chumpich |
| Cucurbitaceae | Disturbance Vegetation | Peten Itza, Rio Hondo, Ria Lagartos1, Ria Lagartos2, Silvituc, Chumpich |
| Flacourtiaceae | Disturbance Vegetation | Peten Itza |
| *Jaquemontia* sp. | Disturbance Vegetation | Peten Itza, Ria Lagartos1, RiaLagartos2 |
| *Justicia campechiana* | Disturbance Vegetation | Chumpich |
| Labiateae | Disturbance Vegetation | Peten Itza, Ria Lagartos1, RiaLagartos2 |
| Loranthaceae type | Disturbance Vegetation | Peten Itza |
| Malvaceae | Disturbance Vegetation | Peten Itza, Rio Hondo, Ria Lagartos1, Ria Lagartos2, Silvituc, chumpich |
| Mimosoideae-*Acacia* | Disturbance Vegetation | Peten Itza, Rio Hondo, Silvituc |
| *Myrica* sp. | Disturbance Vegetation | Ria Lagartos1, Ria Lagartos2, Silvituc, Chumpich |
| Onagraceae | Disturbance Vegetation | Peten Itza, Silvituc |
| Passiflorae | Disturbance Vegetation | Peten Itza, Silvituc |
| *Pilea* sp. | Disturbance Vegetation | Peten Itza |
| Piperaceae | Disturbance Vegetation | Peten Itza |
| Poaceae | Disturbance Vegetation | Peten Itza, Rio Hondo, Ria Lagartos1, Ria Lagartos2, Silvituc, Chumpich |
| *Satureja* sp. | Disturbance Vegetation | Peten Itza |
| Solanaceae | Disturbance Vegetation | Peten Itza, Rio Hondo, Ria Lagartos1, Ria Lagartos2, Chumpich |
| *Trema* sp. | Disturbance Vegetation | Peten Itza |
| Ulmaceae | Disturbance Vegetation | Peten Itza, Silvituc, Chumpich |
| Verbenaceae | Disturbance Vegetation | Ria Lagartos1, Ria Lagartos2 |
|  |  |  |
